# Showing the Bonds—A Subtle but Important Difference in Figure Design that May Alleviate Student Confusion about ATP Hydrolysis

**DOI:** 10.17912/micropub.biology.001540

**Published:** 2025-05-22

**Authors:** Crystal Uminski, Shreya Sujith, Aneesh Nallani, Bryan Armpriest, L. Kate Wright, Dina L. Newman, Mingyu Yang

**Affiliations:** 1 Thomas H. Gosnell School of Life Sciences, Rochester Institute of Technology, Rochester, New York, United States; 2 SUNY Geneseo, Geneseo, New York, United States; 3 Department of Cell and Developmental Biology, University of California, San Diego, San Diego, California, United States

## Abstract

A misconception among biology students is that breaking bonds in adenosine triphosphate (ATP) releases energy. This misconception may be related to imprecise representations of chemical bonding in common diagrams of ATP hydrolysis. We interviewed 33 undergraduate students and randomly assigned them to interpret a figure of ATP hydrolysis that either emphasized bond breaking in the reactants or the formation of new bonds in the products. Students who saw the figure emphasizing bond breaking were more likely to incorrectly classify ATP hydrolysis as endergonic, while students who saw the figure explicitly illustrating bond formation were more likely to use chemically-sound reasoning to describe the reaction.

**Figure 1. Depicting the individual bonds changes students' interpretation of the process and thermodynamics of ATP hydrolysis.**
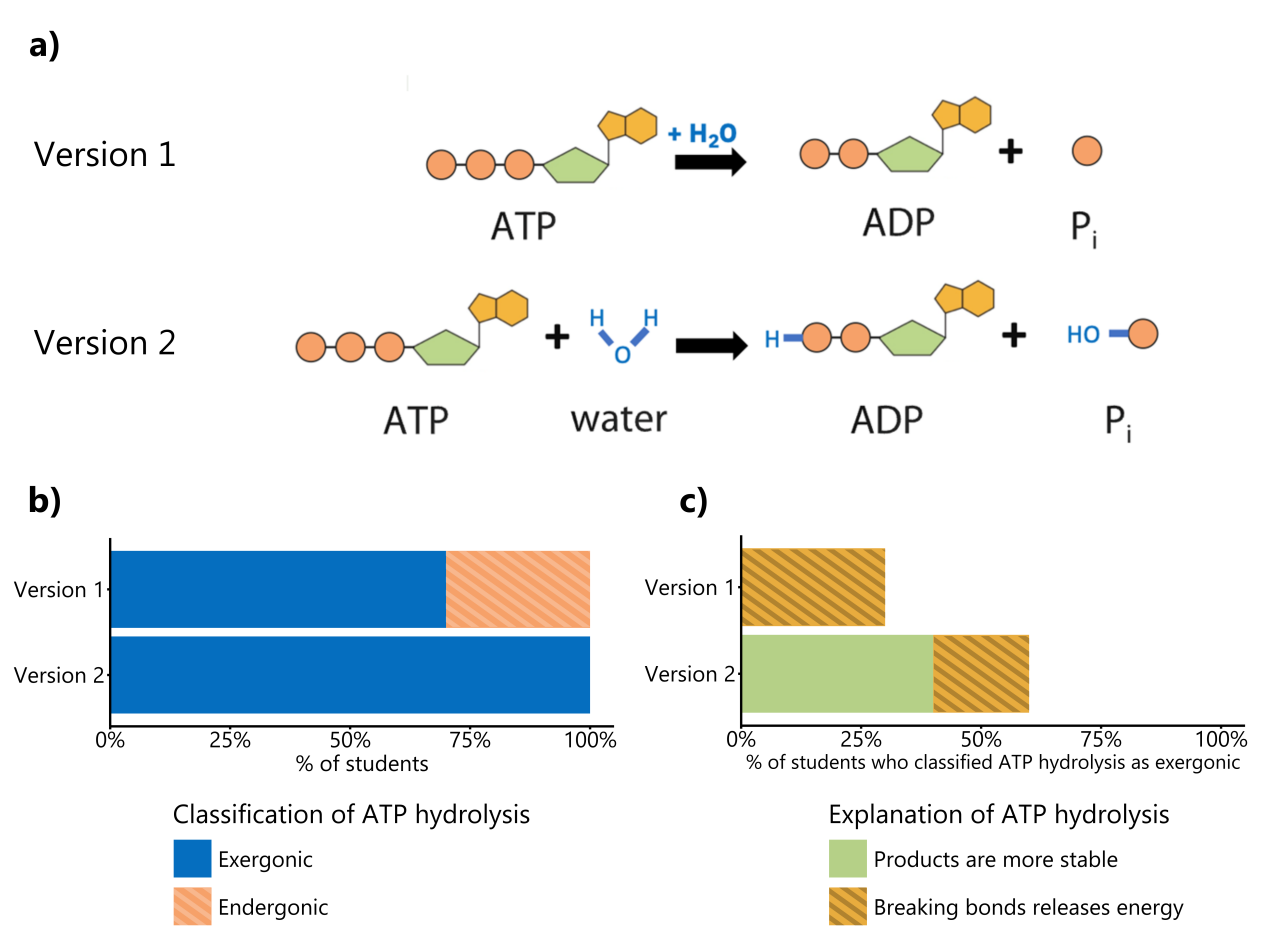
(a) We asked students to discuss ATP hydrolysis after showing them a visual representation of the reaction. Students were randomly assigned to see ATP hydrolysis represented as either *Version 1* or *Version 2* . *Version 1* is a representation of ATP hydrolysis that is commonly found in biology textbooks. *Version 2* is a representation of ATP hydrolysis that explicitly shows which chemical bonds are broken and formed during the reaction. (b) After seeing either *Version 1* or *Version 2* of the ATP hydrolysis reaction, we asked students to classify the reaction as “endergonic” or “exergonic.” Student classifications of ATP hydrolysis as “endergonic” or “exergonic” are partitioned by which version of the reaction they were shown during the interview protocol. (c) After classifying ATP as “endergonic” or “exergonic,” we asked participating students to explain the reasoning behind their answer. Of the students who classified ATP hydrolysis as exergonic, the most common explanations related to the misconception that breaking bonds releases energy or reflected the correct idea that the products in the reaction are more stable. Responses are partitioned by which version of the reaction the student saw during the interview protocol. Student responses unrelated to breaking bonds or product stability were omitted from further analysis.

## Description

Adenosine triphosphate (ATP) is an essential molecule that facilitates energy storage and transfer in cells. Understanding ATP and its role in cellular processes is fundamental to biology. However, many undergraduate students are challenged by the topics related to ATP hydrolysis and its associated energetics—a challenge that is often complicated by the different ways in which science disciplines discuss chemical bond energy (Dreyfus et al., 2014; Kohn et al., 2018). One widespread misunderstanding among biology students is that energy is released directly from breaking the bonds of ATP (Galley, 2004). This misconception, which directly contradicts the chemical principle that breaking bonds requires energy input, is a persistent misunderstanding of ATP hydrolysis that can be perpetuated in college biology courses (Franovic et al., 2023).

We hypothesized that the ubiquity of misconceptions about ATP may be related to how introductory-level biology textbooks typically represent ATP hydrolysis, particularly in the context of chemical bonding. Popular introductory biology textbooks (e.g., Brooker et al., 2011; Freeman et al., 2017; Sadava et al., 2011; Urry et al., 2016) tend to represent ATP hydrolysis as an isolated one-step conversion of adenosine triphosphate to adenosine diphosphate (ADP) and an inorganic phosphate (P _i_ ) accompanied by “energy,” which is often stylized using an halo or explosion-like motif (Yang et al., 2025). Although biology textbooks tend to focus on ATP hydrolysis, ATP is rarely hydrolyzed independently in biological systems and ATP is more often biologically relevant in ATP-coupled processes in which it acts as a phosphorylating agent (Franovic et al., 2023; Liaw & Eisenberg, 1994). While we acknowledge that portraying ATP hydrolysis as an isolated reaction (i.e., “ATP + H _2_ O → ADP + P _i_ ”) can muddle the actual role of ATP in biological systems, ATP hydrolysis is ubiquitously covered in undergraduate biology textbooks (Yang et al., 2025), and we focus here on ATP hydrolysis as an isolated reaction because of its persistence in introductory-level curricula.

Although isolated hydrolysis reactions occur between ATP and water, we previously found that 64% of biology textbook figures omit water as a reactant (i.e., “ATP → ADP + P _i_ ”) (Yang et al., 2025). Instead, textbook diagrams tend to focus on the bond that breaks between the second and third phosphate group in the ATP molecule, often leaving the role of water implicit or unstated. Whereas biology figures often emphasize bond breaking in a single reactant, chemistry textbooks typically depict both bond breaking and formation, focusing on the overall transformation that leads to more stable products (Yang et al., 2025). We hypothesized that the trend of biology textbook figures emphasizing *bond breaking* rather than *bond formation* could potentially hinder students’ ability to comprehend the full mechanism of ATP hydrolysis and the critical role that water plays in this process.

In the present study, we examined how different visual representations of ATP hydrolysis affect student reasoning about the hydrolysis reaction. We conducted semi-structured interviews with 33 students who had completed at least one college biology course and one chemistry course. We used an independent groups design, presenting students with one of two figures of ATP hydrolysis (Figure 1a). In our experimental design, we designed *Version 1* based on common representations of ATP hydrolysis used in biology textbooks (see a complete list of biology textbooks used to inform our figure design in Supplemental Table 1 in Yang et al., 2025). *Version 1* omits water as a reactant and emphasizes bond breaking in the ATP molecule. *Version 2* , which is more akin to how chemistry textbooks portray reactions, includes water as a reactant and illustrates the new bonds formed in the products (see a complete list of chemistry textbooks used to inform our figure design in Supplemental Table 2 in Yang et al., 2025). In our study, we showed each participating student either *Version 1* or *Version 2* and then asked them to use the figure to explain whether ATP hydrolysis was exergonic or endergonic and to describe their reasoning.

We found that most students held partially correct knowledge about ATP hydrolysis and chemical bonding, and that their explanations of the thermodynamics of the hydrolysis reaction varied depending on which version of the reaction representation they saw (Figure 1b). When shown *Version 1* , 30% of students incorrectly classified ATP hydrolysis as an endergonic reaction, yet these same students typically explained hydrolysis using the correct chemical reasoning that breaking bonds requires energy. In contrast, all students shown *Version 2* correctly classified ATP hydrolysis as exergonic.

In both *Version 1* and *Version 2* experimental conditions, despite the majority of students correctly identifying ATP hydrolysis as exergonic, many students invoked incorrect chemical reasoning to explain their choice, explaining that breaking bonds releases energy. To explain their reasoning, one student said: “I assume that the process that we’re talking about is like an exothermic reaction—like it releases energy through the reaction.” Another student justified their choice by stating “Exergonic—it’s just like heat being released, right? Or is energy being released?” Our findings corroborate previous work on student thinking about chemical bonding (Galley, 2004; Johnstone & Mahmoud, 1980; Novick, 1976) and indicate that the role of bond breaking in chemical reactions remains a difficult topic for undergraduate students.

Interestingly, only students who saw *Version 2* accurately discussed product stability as a cause of energy release, which constituted 30% of the responses in this experimental condition (Figure 1c). One student reasoned that “So why ATP hydrolysis is exergonic is because ATP is like somewhat unstable. There’s three phosphates and then taking one off is like to be in a more energetically favorable state.” Another student discussed stability by saying “When you build up to the ATP, you’re storing energy, so it consumes energy to build up and then you would release energy as you break it back down.” Our finding that only students who saw *Version 2* discussed product stability suggests that showing bond formation in visual representations of the reaction may have an impact on student understanding of ATP hydrolysis. Our finding provides preliminary data suggesting that showing bond formation in chemical diagrams may support students in linking the idea of bond stability to energy release.

We also observed that several students described ATP hydrolysis as “exergonic” but gave explanations that were incomplete or difficult to categorize. One student supported their reasoning by stating that ATP hydrolysis was exergonic “because it is breaking down a compound, not creating it.” Another student had intuitive but incorrect logic that “because usually when you break bonds, you’re more likely to have more mobility between the bonds and it could react with other bonds more often—when you keep like a bond together, then it’s not as reactive because it’s connected together, and when you break it apart, you obviously can react.” The range of unexpected explanations for exergonicity highlights the need for additional research into how biology students are thinking about the nature of such reactions in biological contexts. We note that one limitation of our results was that some students were less well-versed in disciplinary vocabulary, such as the terms “endergonic” and “exergonic,” that we used in our interview protocol. While we provided clarifying definitions upon participant request, some students may have felt uncomfortable or embarrassed to request clarification. We suggest future research on topics related to ATP hydrolysis refrain from using jargon, and use phrases such as “energy release” instead of “exergonicity.”

Our preliminary findings here emphasize the importance of clear and accurate representations in conveying biochemical reactions. Showing the bonds in the ATP hydrolysis reaction led more students to correctly classify and explain the energetics of ATP hydrolysis. We encourage introductory biology instructors to examine how traditional curricula on ATP hydrolysis depict energy and chemical bonding in ways that differ from how these concepts are presented in chemistry. We hypothesize that this misalignment may create challenges in student understanding of energy as a crosscutting concept in the sciences. If biology instructors are discussing ATP hydrolysis as an isolated reaction—as is the norm in many biology curricular materials—we suggest taking a brief moment to highlight and describe the chemical bonding in the hydrolysis using terminology consistent with what students encounter in chemistry courses. Small changes in biology teaching that increase the consistency of the representations and descriptions of chemical bonding may help students bridge interdisciplinary thinking about energy.

While showing chemical bond formation can help students think about energetics, additional instructional support may be necessary to help students understand what they are seeing in the diagrams. Some students were correctly classifying the reaction as exergonic for the wrong reasons, highlighting the need for future research to explore how students are conceptualizing visual representations of bonds and how students are understanding bond stability in the context of biological processes. Our results underscore the necessity of consistency in visual representations across instructional materials in biology and chemistry to support students in constructing coherent mental models of energy and chemical reactions.

## Methods

In this study, we interviewed 33 college students from a variety of primarily undergraduate and research intensive institutions. All study participants had completed at least one year of introductory biology and chemistry coursework. We recruited study participants through flyers, in-class advertisements, and personal networks at an R2 private research institution. The participants were provided with informed consent regarding the study, and were compensated for their time with a $10 Amazon gift card. We conducted interviews over the video conferencing platform Zoom.

We used a semi-structured interview protocol in which we asked participants a series of questions designed to determine their understanding of ATP and ATP hydrolysis. We randomly assigned participants to one of two treatment conditions in which the interview protocol differed only in the way that the ATP hydrolysis was visually represented (Figure 1a). There were 17 students who were shown *Version 1* and 16 students who were shown *Version 2* .

We asked students to interpret the visual representation of ATP hydrolysis (e.g., “What information can you get from this image?” and “What are some strengths and weaknesses of this image in depicting this process?”) and then asked them to explain their reasoning. We asked additional questions on the energetics of the ATP hydrolysis reaction after the participant had viewed the diagram (e.g., “Is ATP hydrolysis an exergonic or endergonic reaction? Why?” and “How is ATP often used to drive reactions?”).

We recorded and transcribed all the interviews for analysis. We coded the data using an emergent coding scheme to identify themes and patterns in student responses to the interview protocol. One researcher developed a codebook, and a second coder validated it by independently analyzing the transcripts. Discrepancies were discussed and resolved to refine the codebook, and subsequent interviews were analyzed collaboratively by the second and a third coder.

This research was approved and classified as exempt from human-subjects review by the Rochester Institute of Technology Institutional Review Board (protocol #03053023).
